# Characterization of the safety notes of pharmaceutical products issued by two regulatory authorities from Latin America

**DOI:** 10.17843/rpmesp.2026.432.15489

**Published:** 2026-06-26

**Authors:** Katheryn Pérez-Salazar, Marilena Yataco-Camarena, L. Yesenia Rodríguez-Tanta

**Affiliations:** 1 Grupo Peruano de Investigación en Medicamentos, Políticas y Servicios Farmacéuticos (GPIMPSF), Carrera de Farmacia y Bioquímica, Universidad Científica del Sur, Lima, Peru

**Keywords:** Chile, Peru

## Abstract

Pharmaceutical product safety notes (PPSNs) are regulatory communications that disseminate updated information on the risks of pharmaceutical products (PPs). This study characterized the PPSNs issued by the Direccion General de Medicamentos, Insumos y Drogas (Peru) and the Instituto de Salud Publica de Chile (ISP) between 2014 and 2023. A retrospective analysis of PPSNs published on their websites was conducted. Those describing adverse drug reactions (ADRs) were included. Data were extracted on the type of ADRs, regulatory actions, sources of evidence, and reasons for issuance. Of 746 PPSNs identified, 153 met the criteria (100 Peru, 53 Chile), with a monthly average of 0.83 and 0.44, respectively. Differences in content and PPs were observed. Both agencies based their PPSNs mainly on alerts from the U.S. Food and Drug Administration. The most frequent action was the modification of the technical data sheet; the ISP additionally implemented monitoring and restrictions. In conclusion, PPSNs depend on external alerts, which evidences the need to strengthen local pharmacovigilance and evaluate their impact on the safety of PPs.

## INTRODUCTION

Pharmaceutical product safety notes (PPSNs) are pharmacovigilance tools developed by drug regulatory authorities (DRAs) to inform about risks associated with pharmaceutical products (PPs) and promote their safe use ^1^^,^^2^. These notices are based on various sources of evidence, including observational studies, clinical trials, and pharmacovigilance systems, and are disseminated on the official websites of the DRAs.

Although PPSNs may receive different denominations depending on the country, their objective is common. In Chile, the Public Health Institute (ISP) defines them as documents that describe risks and offer recommendations for the safe use of PPs ^3^, while, in Peru, the General Directorate of Medicines, Supplies and Drugs (DIGEMID) calls them alerts and includes information about adverse drug reactions (ADRs), at-risk populations, and preventive measures ^4^.

Previous studies have evidenced their impact on health regulation. In Europe, between 32% and 45% of alerts generated changes in the technical data sheet and up to 9% drug withdrawals ^5^^,^^6^. Internationally, a predominance of central nervous system (23%) and cardiovascular (21%) drugs has been reported ^7^. However, there are no studies in Latin America. Therefore, this study characterizes the PPSNs issued by DIGEMID (Peru) and ISP (Chile) between 2014 and 2023.

**KEY MESSAGES**

Motivation for the study. Pharmaceutical product safety notes (PPSNs) communicate information about new drug risks and provide regulatory and clinical recommendations, contributing to safer prescription.

Main findings. Differences in the content of PPSNs were observed between Peru and Chile. In common, the most frequent regulatory action was the modification of the technical data sheet, based mainly on international alerts, rather than local evidence, which reflects alignment with global standards, but also limitations of local pharmacovigilance systems.

Public health implications. To maximize their impact, PPSNs require stronger pharmacovigilance systems. These advancements would allow for improved patient safety, while also strengthening the credibility and independence of regulatory authorities.

## THE STUDY

A retrospective analysis of the PPSNs published on the websites of the ADRs DIGEMID (Peru) and ISP (Chile) between January 1, 2014, and December 31, 2023, was carried out. PPSNs that described ADRs, the population at risk, and clinical or regulatory recommendations were included. Those related to quality problems, counterfeiting, marketing authorization, or illegal marketing were excluded.

Two reviewers independently evaluated the PPSNs by reviewing titles and full content, applying predefined eligibility criteria; discrepancies were resolved with a third reviewer. Subsequently, data extraction was performed using a standardized form that collected information on the publication date, the issuing country, the drugs involved according to the International Nonproprietary Name (INN), their pharmacological classification (first level of ATC) ^8^ and clinical indication. The presence of PPs in the National Essential Medicines List (LNME) ^9^^,^^10^ and in the clinical setting of use was also recorded.

Regarding safety aspects, the type of ADR was recorded, classified according to the MedDRA System Organ Classes (SOC) ^(^^11^, as well as its severity as described in each PPSNs. The source of justification was identified, classifying it as primary (based on local reports) or secondary (derived from alerts from high-vigilance regulatory agencies), and, in the latter case, the type of evidence used was characterized, including clinical trials, observational studies, meta-analyses, spontaneous notifications, preclinical studies, or combinations. Additionally, the recurrence of PPSNs during the study period was evaluated.

Likewise, the regulatory and clinical actions described in the PPSNs were identified and extracted. Regulatory actions were defined as measures adopted by the DRAs due to PPs safety risks (product information modification, restrictions, market withdrawal, additional pharmacovigilance, or risk management plans). Clinical actions included recommendations for medical practice, such as monitoring, patient education, treatment suspension, or the use of therapeutic alternatives.

Descriptive statistics (frequencies and percentages) were used. Statistical analyses were performed in R version 4.4.3.

The study used publicly available information and did not involve human participants or personal data, so approval was not requested from a Research Ethics Committee.

## FINDINGS

**Figure 1:**
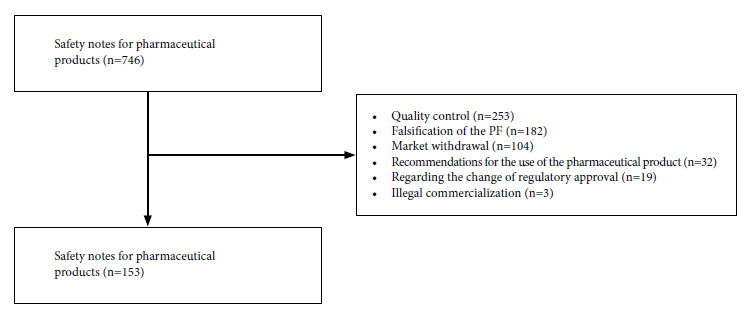
Flowchart summarizing the identification of pharmaceutical product safety notes in Peru and Chile (2014-2023).

746 PPSNs were identified and after applying the exclusion criteria, we included 153 PPSNs (100 from DIGEMID and 53 from ISP). The exclusion reasons are detailed in figure 1.

**Figure 2:**
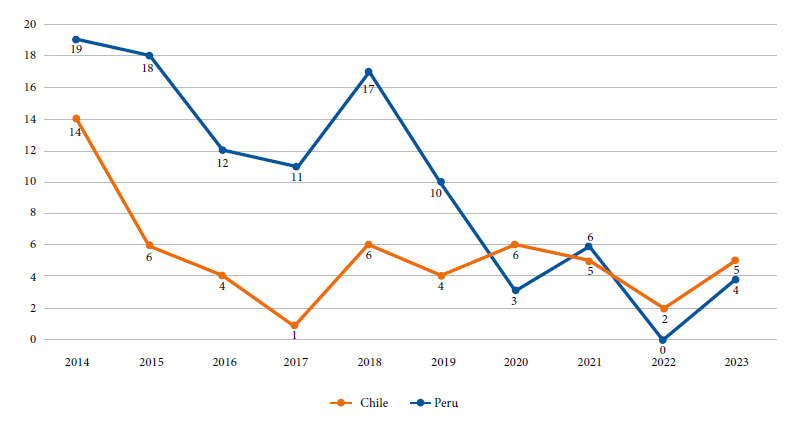
Annual trends in publication of pharmaceutical product safety notes in Peru and Chile (2014-2023).

Figure 2 shows the annual publication trends. In Peru, PPSNs varied considerably, decreasing from 19 in 2014 to 0 in 2022, with a slight increase in 2021 (4 NSPF). In Chile, the trend was more stable, with a decrease from 14 in 2014 to 1 in 2017, followed by an increase to 5 in 2023.

DIGEMID issued 100 PPSNs which reported 176 ADRs; about 47% described between two and five events, with a maximum of five per PPSNs. The most frequent ADRs were Stevens-Johnson syndrome (SJS) (n=8), toxic epidermal necrolysis (TEN), heart failure, hepatotoxicity and fetal damage (n=5 each). According to MedDRA, skin and subcutaneous tissue disorders (n=24) and vascular disorders (n=23) predominated.

The ISP reported 88 ADRs; 38% of the PPSNs included between two and nine events. The most frequent were hepatotoxicity and cancer (n=6 each), followed by osteonecrosis of the jaw, progressive multifocal leukoencephalopathy, and heart failure (n=3 each). According to MedDRA, nervous system disorders (n=12) and cardiac disorders (n=11) predominated (table 1).

**Table 1:** Adverse drug reactions reported in pharmaceutical product safety notes according to MedDRA System Organ Class classification.

Adverse drug reactions by organ and system classification	Peru (n=176)		Chile (n=88)	
	n	%	n	%
Vascular disorders	23	13.1	5	5.7
Disorders of the skin and subcutaneous tissue	24	13.6	3	3.4
Cardiac disorders	16	9.1	11	12.5
Disorders of the nervous system	13	7.4	12	13.6
Psychiatric disorders	14	8.0	9	10.2
Infections and infestations	13	7.4	1	1.1
Congenital, familial, and genetic disorders	11	6.3	2	2.3
Musculoskeletal and connective tissue disorders	9	5.1	6	6.8
Disorders of the blood and the lymphatic system	9	5.1	4	4.6
Renal and urinary disorders	7	4.0	3	3.4
Gastrointestinal disorders	7	4.0	3	3.4
Respiratory, thoracic and mediastinal disorders	6	3.4	1	1.1
Benign, malignant, and unspecified neoplasms	6	3.4	7	7.9
Hepatobiliary disorders	5	2.8	7	7.9
Disorders of the reproductive system and of the breast	5	2.8	5	5.7
Disorders of the immune system	3	1.7	2	2.3
Endocrine disorders	1	0.6	3	3.4
Ocular disorders	1	0.6	1	1.1
Injuries, Poisonings and Complications of Procedures	1	0.6	0	0.0
Metabolic and Nutritional Disorders	1	0.6	3	3. 4
General disorders and administration site reactions	1	0.6	0	0.0

ADRs: adverse drug reactions, SOC: System Organ Class.

In Peru, 146 PPs were identified in the PPSNs; according to the ATC classification, 22% corresponded to nervous system medications (N), 21% to antineoplastic and immunomodulating agents (L), and 15% to musculoskeletal system drugs (M). The most frequent PPs were SGLT-2 inhibitors (canagliflozin, dapagliflozin, ATC A10BK) and ibuprofen (M01AE01). 46% of the PPs were included in Peru's LNME. In Chile, 68 PPs were identified; 19% corresponded to alimentary tract and metabolism medications (A), another 19% to systemic anti-infectives (J), and 15% to nervous system drugs (N). The most reported were DPP-4 inhibitors (ATC A10BH01). 28% of the PPs were found in Chile's LNME (table 2).

**Table 2:** Medicines classified according to the anatomical, therapeutic, and chemical (ATC) classification by pharmaceutical product safety notes.

Anatomical, Therapeutic, and Chemical Classification Code	Peru (n=100)	Chile (n=53)
	n (%)	n (%)
N	22 (22.0)	8 (15.1)
L	21 (21.0)	7 (13.2)
M	15 (15.0)	5 (9.4)
A	13 (13.0)	10 (18.9)
J	9 (9.0)	10 (18.9)
C	4 (4.0)	3 (5.7)
B	2 (2.0)	4 (7.5)
R	4 (4.0)	1 (1.9)
D	2 (2.0)	2 (3.8)
V	2 (2.0)	2 (3.8)
G	2 (2.0)	1 (1.9)
D / G	1 (1.0)	0 (0.0)
H	1 (1.0)	0 (0.0)
P	1 (1.0)	0 (0.0)
S	1 (1.0)	0 (0.0)

A: alimentary tract and metabolism, B: blood and hematopoietic organs, C: cardiovascular system, D: dermatologicals, G: genitourinary system and sex hormones, H: systemic hormonal preparations (excluding sex hormones and insulins), J: antiinfectives for systemic use, L: antineoplastic and immunomodulating agents, M: musculoskeletal system, N: nervous system, P: antiparasitic products, insecticides and repellents, R: respiratory system, S: sensory organs, V: various, D/G: combination of dermatologicals and genitourinary system.

Most PPSNs was based on alerts from the U.S. Food and Drug Administration (FDA). Exceptionally, in Peru, an NSPF was identified based on a local report of SJS associated with antituberculosis drugs, reported to DIGEMID ^12^. Upon analyzing the original technical evidence used by the FDA to generate these alerts, it was observed that 40.4% of the notes in Peru and 39.6% in Chile were based on international alerts based on spontaneous reporting of ADRs (table 3).

**Table 3:** Sources of evidence, regulatory and clinical actions derived from pharmaceutical products safety notes in Peru and Chile.

Variable	Category	Peru (n=100)	Chile (n=53)
		n (%)	n (%)
Source of evidence used^a^	Spontaneous Notification of ADRs	40 (40.4)	21 (39.6)
	Observational study + spontaneous reporting	33 (33.3)	17 (32.1)
	Clinical trials + spontaneous reporting	7 (7.1)	10 (18.9)
	Clinical trials	14 (14.1)	0 (0.0)
	Meta-analysis	3 (3.1)	3 (5.7)
	Observational study	2 (2.0)	0 (0.0)
	Preclinical study	0 (0.0)	2 (3.8)
Regulatory actions	Modification of the SmPC	90 (90.0)	10 (18.9)
	Not specified	9 (9.0)	33 (62.3)
	Restriction of use	(1.0)	2 (3.8)
	Continuous Monitoring	0 (0.0)	4 (7.6)
	Request by RMP for MAH	0 (0.0)	1 (1.9)
	Preventive withdrawal	0 (0.0)	1 (1.9)
	Comprehensive Safety Evaluation	0 (0.0)	2 (3.8)
Clinical actions	Monitor, evaluate, inform the patient	64 (64.0)	33 (62.3)
	Monitor and suspend if necessary	28 (28.0)	14 (26.4)
	Monitor and consider alternative	7 (7.0)	5 (9.4)
	Restriction of use	1 (1.0)	1 (1.9)

ADRs: adverse drug reactions, PPSNs: pharmaceutical product safety notes, SmPC: summary of product characteristics, RMP: risk management plan, MAH: marketing authorization holder.

a In Peru, the percentages of the variable "Source of evidence used" were calculated using a denominator of 99, because one PPSNs was based on local notification.

90% of DIGEMID's notes reported at least one measure, compared to 37.7% of ISP's. The most frequent modification in both was that of the technical data sheet and the product insert. However, the ISP included continuous monitoring of methylphenidate and diacerein (due to the risk of priapism and severe diarrhea), as well as of the ChAdOx1 vaccine against COVID-19 (due to possible Guillain-Barré syndrome and systemic capillary leak). Furthermore, it required a risk management plan for gadolinium-based contrast agents due to their cerebral accumulation ^13^. DIGEMID did not report requirements for continuous monitoring, additional pharmacovigilance measures, or preventive drug withdrawals.

The most frequent categories of clinical recommendations were «Monitor, evaluate, inform the patient; consider dose and vulnerable/specific populations» and «Monitor, evaluate and, if necessary, discontinue treatment». Restrictions on use were also recorded: in Peru, topical lidocaine for pediatric teething was restricted in 2015 due to serious risks (convulsions and death), and in Chile, hydroxyethyl starch was restricted in 2018 due to its association with kidney damage and increased mortality in patients with septicemia.

## DISCUSSION

Between 2014 and 2023, DIGEMID issued 100 PPSNs and ISP 53, approximately 0.83 per month in Peru and 0.44 per month in Chile. Peaks were observed in 2014 and 2018, followed by a decrease since 2019, which reached zero in Peru in 2022. This reduction could be related to the COVID-19 pandemic, which reoriented regulatory resources towards quality control and surveillance of falsified medicines ^14^^,^^15^.

In Peru, PPSNs focused primarily on dermatological ADRs (SJS, TEN), while in Chile, hepatotoxicity and cancer predominated. These differences could be explained by variations in drug use patterns, in pharmacovigilance, and in population factors. In Peru, SGLT-2 inhibitors stood out, and in Chile, DPP-4 inhibitors, reflecting their increasing use in type 2 diabetes. This increase has been associated with an increase in reported ADRs, such as genital infections, ketoacidosis, and rare events with SGLT-2, and pancreatitis and hypersensitivity with DPP-4 ^16^^,^^17^, which highlights the need to strengthen active pharmacovigilance.

Differences in PPSNs were observed between Peru and Chile according to the ATC classification, which could reflect variations in therapeutic practices and safety priorities. In Peru, most PPSNs focused on nervous system (N) medications (22%), including antidepressants, antipsychotics, and anticonvulsants, associated with dermatological, neurological, and psychiatric ADRs, which is consistent with what has been reported in other countries ^7^. In Chile, the Alimentary Tract and Metabolism (A) group (19%) predominated, which includes drugs for gastrointestinal and metabolic disorders such as type 2 diabetes and obesity. These differences could reflect variations in disease burden and treatment patterns, underscoring the need for pharmacovigilance adapted to the local context.

When evaluating the presence of the medications described in the PPSNs in the LNME, 46% of the PPs in Peru and 27% in Chile were included in their respective lists. While essential medicines are safe and effective, their extended use increases the probability of ADRs, which makes their continuous monitoring indispensable ^9^^,^^18^. The Peruvian LNME currently includes 434 PPs, of which four are under intensive pharmacovigilance ^9^. Our findings identified other essential medicines with the potential to cause serious ADRs, so we recommend that DIGEMID expand its active pharmacovigilance programs to include high-risk and high-consumption drugs.

The PPSNs issued by DIGEMID and ISP were based on FDA alerts and, only in Peru, an alert based on local pharmacovigilance was identified. This could suggest limitations in local pharmacovigilance systems, mainly, to generate safety signals from national data. Likewise, the evidence supporting international alerts comes from spontaneous reports of ADRs and post-marketing observational studies. Although clinical trials are key to evaluating efficacy, pharmacoepidemiological studies and ADRs reports are fundamental for detecting risks in broad populations ^19^^,^^20^. This highlights the need to strengthen reporting systems and incorporate pharmacoepidemiological studies in Peru and Chile, in order to improve regulatory decision-making and patient safety.

Regarding regulatory actions, although product datasheet and insert modifications predominated in both countries, the ISP implemented additional measures, such as continuous monitoring of specific drugs (methylphenidate and vaccines like ChAdOx1 and Ad26.COV2) and the request for risk management plans for gadolinium due to its possible cerebral accumulation. These actions reflect a more preventive and proactive approach than that of DIGEMID, which tends to act more reactively, responding to events that have already occurred or external alerts. These differences could be explained by variations in regulatory frameworks, access to evidence, and resource availability.

This study presents some limitations. PPSNs, being concise documents, do not include all technical provisions (e.g., periodic safety reports or risk management plans), which could underestimate regulatory actions. Additionally, it was not possible to determine whether PPs withdrawals were definitive or temporary. However, the study has relevant strengths: it is, to our knowledge, the first descriptive-comparative analysis of PPSNs in Latin American countries, which evaluates not only the frequency of alerts, but also the traceability of evidence (international vs. local) and inclusion in the LNME, offering a comprehensive view of the reliance on international surveillance and its impact on regional public health.

Between 2014 and 2023, 153 PPSNs related to ADRs were issued, with an average of approximately two per month in Peru and one in Chile. Differences were identified in their content, particularly in the ADRs and the distribution by ATC. Most PPSNs were based on FDA alerts, and the modification of the technical data sheet was the main regulatory measure. However, the ISP implemented additional actions, such as continuous monitoring, use restrictions, and risk management plans. These findings highlight the need to strengthen local pharmacovigilance in both countries, especially the spontaneous reporting of ADRs.
