# Supplementary material for: Characterization of the safety notes of pharmaceutical products issued by two regulatory authorities from Latin America
Source: Rev Peru Med Exp Salud Publica. 2026 Jun 26;43(2):231–7. doi: 10.17843/rpmesp.2026.432.15489 (PMC13456923; doi:10.17843/rpmesp.2026.432.15489)
Supplement: Translation

# Translation: Caracterización de las notas de seguridad de productos farmacéuticos emitidas por dos autoridades reguladoras de América Latina

**Authors:** Katheryn Pérez-Salazar, Marilena Yataco-Camarena, L. Yesenia Rodríguez-Tanta

**Affiliations:** 1 Grupo Peruano de Investigación en Medicamentos, Políticas y Servicios Farmacéuticos (GPIMPSF), Carrera de Farmacia y Bioquímica, Universidad Científica del Sur, Lima, Perú

**Keywords:** Farmacovigilancia, América Latina, Chile, Perú

## Abstract

Las notas de seguridad de productos farmacéuticos (NSPF) son comunicaciones regulatorias que difunden información actualizada sobre riesgos de productos farmacéuticos (PF). Este estudio caracterizó las NSPF emitidas por la Dirección General de Medicamentos, Insumos y Drogas (Perú) y el Instituto de Salud Pública de Chile (ISP) entre 2014 y 2023. Se realizó un análisis retrospectivo de NSPF publicadas en sus sitios web. Se incluyeron aquellas que describían reacciones adversas a medicamentos (RAMs). Se extrajeron datos sobre tipo de RAMs, acciones regulatorias, fuentes de evidencia y razones de emisión. De 746 NSPF identificadas, 153 cumplieron criterios (100 Perú, 53 Chile), con un promedio mensual de 0,83 y 0,44, respectivamente. Se observaron diferencias en contenido y PF. Ambas agencias basaron sus NSPF principalmente en alertas de la U.S. Food and Drug Administration. La acción más frecuente fue la modificación de la ficha técnica; el ISP además, implementó monitoreo y restricciones. En conclusión, las NSPF dependen de alertas externas, lo que evidencia la necesidad de fortalecer la farmacovigilancia local y evaluar su impacto en la seguridad de los PF.

## INTRODUCCIÓN

Las notas de seguridad de productos farmacéuticos (NSPF) son herramientas de farmacovigilancia elaboradas por las autoridades regulatorias de medicamentos (ARM) para informar sobre los riesgos asociados a productos farmacéuticos (PF) y promover su uso seguro ^1^^,^^2^. Estas notas se basan en diversas fuentes de evidencia, incluidos estudios observacionales, ensayos clínicos y sistemas de farmacovigilancia, y se difunden en los sitios web oficiales de las ARM.

Aunque las NSPF pueden recibir distintas denominaciones según el país, su objetivo es común. En Chile, el Instituto de Salud Pública (ISP) las define como documentos que describen riesgos y ofrecen recomendaciones para el uso seguro de PF ^3^, mientras que, en Perú, la Dirección General de Medicamentos, Insumos y Drogas (DIGEMID) las denomina alertas e incluye información sobre reacciones adversas a medicamentos (RAMs), poblaciones en riesgo y medidas preventivas ^4^.

Estudios previos han evidenciado su impacto en la regulación sanitaria. En Europa, entre el 32% y 45% de las alertas generaron cambios en la ficha técnica y hasta el 9% retiros de medicamentos ^5^^,^^6^. A nivel internacional, se ha reportado un predominio de medicamentos del sistema nervioso central (23%) y cardiovasculares (21%) ^7^. Sin embargo, no existen estudios en América Latina. Por ello, este estudio caracteriza las NSPF emitidas por DIGEMID (Perú) e ISP (Chile) entre 2014 y 2023.

**MENSAJES CLAVE**

Motivación del estudio. Las notas de seguridad de productos farmacéuticos (NSPF) comunican información sobre nuevos riesgos de los medicamentos y brindan recomendaciones regulatorias y clínicas, contribuyendo a una prescripción más segura.

Principales hallazgos. Se observaron diferencias en el contenido de las NSPF entre Perú y Chile. En común, la acción regulatoria más frecuente fue la modificación de la ficha técnica, basada principalmente en alertas internacionales, más que en evidencia local, lo que refleja alineación con estándares globales, pero también limitaciones de los sistemas de farmacovigilancia locales.

Implicancias en salud pública. Para maximizar su impacto, las NSPF requieren sistemas de farmacovigilancia más sólidos. Estos avances permitirían mejorar la seguridad del paciente, al mismo tiempo que fortalecerían la credibilidad e independencia de las autoridades regulatorias.

## EL ESTUDIO

Se realizó un análisis retrospectivo de las NSPF publicadas en los sitios web de las ARMs DIGEMID (Perú) e ISP (Chile) entre el 1 de enero de 2014 y el 31 de diciembre de 2023. Se incluyeron NSPF que describían RAMs, la población en riesgo y recomendaciones clínicas o regulatorias. Se excluyeron aquellas relacionadas con problemas de calidad, falsificación, registro sanitario o comercialización ilegal.

Dos revisores evaluaron de forma independiente las NSPF mediante la revisión de títulos y contenido completo, aplicando criterios de elegibilidad predefinidos; las discrepancias se resolvieron con una tercera revisora. Posteriormente, la extracción de datos se realizó mediante un formulario estandarizado que recopiló información sobre la fecha de publicación, el país emisor, los medicamentos involucrados según la Denominación Común Internacional (DCI), su clasificación farmacológica (primer nivel de ATC) ^8^ e indicación clínica. También se registró la presencia de los PF en la Lista Nacional de Medicamentos Esenciales (LNME) ^9^^,^^10^ y en el ámbito clínico de uso.

En cuanto a los aspectos de seguridad, se registró el tipo de RAM, clasificada según las Clases de Órganos y Sistemas (SOC) de MedDRA ^11^, así como su gravedad según lo descrito en cada NSPF. Se identificó la fuente de justificación, clasificándola como primaria (basada en reportes locales) o secundaria (derivada de alertas de agencias regulatorias de alta vigilancia), y, en este último caso, se caracterizó el tipo de evidencia utilizada, incluyendo ensayos clínicos, estudios observacionales, metaanálisis, notificaciones espontáneas, estudios preclínicos o combinaciones. Además, se evaluó la reiteración de las NSPF durante el periodo de estudio.

Asimismo, se identificaron y extrajeron las acciones regulatorias y clínicas descritas en las NSPF. Las acciones regulatorias se definieron como medidas adoptadas por las ARM ante riesgos de seguridad de PF (modificación de la ficha técnica, restricciones, retiro del mercado, farmacovigilancia adicional o planes de gestión de riesgos). Las acciones clínicas incluyeron recomendaciones para la práctica médica, como el monitoreo, la educación del paciente, la suspensión del tratamiento o el uso de alternativas terapéuticas.

Se utilizaron estadísticas descriptivas (frecuencias y porcentajes). Los análisis estadísticos se realizaron en R versión 4.4.3.

El estudio utilizó información de acceso público y no involucró participantes humanos ni datos personales, por lo que no se solicitó la aprobación de un Comité de Ética en Investigación.

## HALLAZGOS

Se identificaron 746 NSPF y tras aplicar los criterios de exclusión, incluimos 153 NSPF (100 de DIGEMID y 53 del ISP). Las razones de exclusión se detallan en la figura 1.

**Figura 1:** Diagrama de flujo que resume la identificación de las notas de seguridad de productos farmacéuticos en Perú y Chile (2014-2023).

La figura 2 muestra las tendencias anuales de publicación. En Perú, las NSPF variaron considerablemente, pasando de 19 en 2014 a 0 en 2022, con un leve incremento en 2021 (4 NSPF). En Chile, la tendencia fue más estable, con una disminución de 14 en 2014 a 1 en 2017, seguida de un incremento hasta 5 en 2023.

**Figura 2:** Tendencias anuales de publicación de notas de seguridad de productos farmacéuticos en Perú y Chile (2014-2023).

La DIGEMID emitió 100 NSPF que reportaron 176 RAMs; cerca del 47% describió entre dos y cinco eventos, con un máximo de cinco por NSPF. Las RAMs más frecuentes fueron el síndrome de Stevens-Johnson (SSJ) (n=8), la necrólisis epidérmica tóxica (NET), insuficiencia cardíaca, hepatotoxicidad y daño fetal (n=5 cada una). Según MedDRA, predominaron los trastornos de la piel y tejido subcutáneo (n=24) y vasculares (n=23).

El ISP reportó 88 RAMs; el 38% de las NSPF incluyó entre dos y nueve eventos. Las más frecuentes fueron hepatotoxicidad y cáncer (n=6 cada una), seguidas de osteonecrosis de la mandíbula, leucoencefalopatía multifocal progresiva e insuficiencia cardíaca (n=3 cada una). Según MedDRA, predominaron los trastornos del sistema nervioso (n=12) y cardíaco (n=11) (tabla 1).

**Tabla 1:** Reacciones adversas a medicamentos reportadas en las notas de seguridad de productos farmacéuticos según la clasificación por Sistemas de órganos y sistemas de MedDRA.

Reacciones adversas a medicamentos según clasificación por órganos y sistemas	Perú (n=176)		Chile (n=88)	
	n	%	n	%
Trastornos vasculares	23	13,1	5	5,7
Trastornos de la piel y del tejido subcutáneo	24	13,6	3	3,4
Trastornos cardiacos	16	9,1	11	12,5
Trastornos del sistema nervioso	13	7,4	12	13,6
Trastornos psiquiátricos	14	8,0	9	10,2
Infecciones e infestaciones	13	7,4	1	1,1
Trastornos congénitos, familiares y genéticos	11	6,3	2	2,3
Trastornos musculoesqueléticos y del tejido conectivo	9	5,1	6	6,8
Trastornos de la sangre y del sistema linfático	9	5,1	4	4,6
Trastornos renales y urinarios	7	4,0	3	3,4
Trastornos gastrointestinales	7	4,0	3	3,4
Trastornos respiratorios, torácicos y mediastínicos	6	3,4	1	1,1
Neoplasias benignas, malignas y no especificadas	6	3,4	7	7,9
Trastornos hepatobiliares	5	2,8	7	7,9
Trastornos del aparato reproductor y de la mama	5	2,8	5	5,7
Trastornos del sistema inmunológico	3	1,7	2	2,3
Trastornos endocrinos	1	0,6	3	3,4
Trastornos oculares	1	0,6	1	1,1
Lesiones, intoxicaciones y complicaciones de procedimientos	1	0,6	0	0,0
Trastornos metabólicos y nutricionales	1	0,6	3	3,4
Trastornos generales y alteraciones en el lugar de administración	1	0,6	0	0,0

RAM: reacciones adversas a medicamentos, SOC: clasificación por sistemas de órganos y aparatos.

En Perú, se identificaron 146 PF en las NSPF; según la clasificación ATC, el 22% correspondió a medicamentos del sistema nervioso (N), el 21% a agentes antineoplásicos e inmunomoduladores (L) y el 15% a fármacos del sistema musculoesquelético (M). Los PF más frecuentes fueron los inhibidores de SGLT-2 (canagliflozina, dapagliflozina, ATC A10BK) y el ibuprofeno (M01AE01). El 46% de los PF estaba incluido en la LNME de Perú. En Chile, se identificaron 68 PF; el 19% correspondió a medicamentos del tracto alimentario y metabolismo (A), otro 19% a antiinfecciosos sistémicos (J) y el 15% a fármacos del sistema nervioso (N). Los más reportados fueron los inhibidores de la DPP-4 (ATC A10BH01). El 28% de los PF se encontraba en la LNME de Chile (tabla 2).

**Tabla 2:** Medicamentos clasificados según la clasificación anatómica, terapéutica y química (ATC) por notas de seguridad de productos farmacéuticos.

Código de clasificación anatómica, terapéutica y química	Perú (n=100)	Chile (n=53)
	n (%)	n (%)
N	22 (22,0)	8 (15,1)
L	21 (21,0)	7 (13,2)
M	15 (15,0)	5 (9,4)
A	13 (13,0)	10 (18,9)
J	9 (9,0)	10 (18,9)
C	4 (4,0)	3 (5,7)
B	2 (2,0)	4 (7,5)
R	4 (4,0)	1 (1,9)
D	2 (2,0)	2 (3,8)
V	2 (2,0)	2 (3,8)
G	2 (2,0)	1 (1,9)
D / G	1 (1,0)	0 (0,0)
H	1 (1,0)	0 (0,0)
P	1 (1,0)	0 (0,0)
S	1 (1,0)	0 (0,0)

A: tracto alimentario y metabolismo, B: sangre y órganos hematopoyéticos, C: sistema cardiovascular, D: dermatológicos, G: sistema genitourinario y hormonas sexuales, H: preparados hormonales sistémicos (excluyendo hormonas sexuales e insulinas), J: antiinfecciosos de uso sistémico, L: agentes antineoplásicos e inmunomoduladores, M: sistema musculoesquelético, N: sistema nervioso, P: productos antiparasitarios, insecticidas y repelentes, R: sistema respiratorio, S: órganos de los sentidos, V: varios, D/G: combinación de dermatológicos y sistema genitourinario.

La mayoría de NSPF se basó en alertas de la U.S. Food and Drug Administration (FDA). De forma excepcional, en Perú se identificó una NSPF basada en un reporte local de SSJ asociado a fármacos antituberculosos, notificado a la DIGEMID ^12^. Al analizar la evidencia técnica original utilizada por la FDA para generar dichas alertas, se observó que el 40,4% de las notas en Perú y el 39,6% en Chile se sustentaron en alertas internacionales basadas en la notificación espontánea de RAM (tabla 3).

**Tabla 3:** Fuentes de evidencia, acciones regulatorias y clínicas derivadas de las notas de seguridad de productos farmacéuticos en Perú y Chile.

Variable	Categoría	Perú (n=100)	Chile (n=53)
		n (%)	n (%)
Fuente de evidencia utilizada^a^	Notificación espontánea de RAMs	40 (40,4)	21 (39,6)
	Estudio observacional + notificación espontánea	33 (33,3)	17 (32,1)
	Ensayos clínicos + notificación espontánea	7 (7,1)	10 (18,9)
	Ensayos clínicos	14 (14,1)	0 (0,0)
	Meta-análisis	3 (3,1)	3 (5,7)
	Estudio observacional	2 (2,0)	0 (0,0)
	Estudio preclínico	0 (0,0)	2 (3,8)
Acciones regulatorias	Modificación del SmPC	90 (90,0)	10 (18,9)
	No especifica	9 (9,0)	33 (62,3)
	Restricción de uso	1 (1,0)	2 (3,8)
	Monitoreo continuo	0 (0,0)	4 (7,6)
	Solicitud de PGR al TAC	0 (0,0)	1 (1,9)
	Retiro preventivo	0 (0,0)	1 (1,9)
	Evaluación integral de seguridad	0 (0,0)	2 (3,8)
Acciones clínicas	Monitorear, evaluar, informar al paciente	64 (64,0)	33 (62,3)
	Monitorear y suspender si es necesario	28 (28,0)	14 (26,4)
	Monitorear y considerar alternativa	7 (7,0)	5 (9,4)
	Restricción de uso	1 (1,0)	1 (1,9)

RAM: reacciones adversas a medicamentos, NSPF: notas de seguridad de productos farmacéuticos, SmPC: resumen de características del producto, PGR: plan de gestión de riesgos, TAC: titular de autorización de comercialización.

a En Perú, los porcentajes de la variable “Fuente de evidencia utilizada” se calcularon utilizando un denominador de 99, debido a que una NSPF se basó en notificación local.

El 90% de las notas de DIGEMID reportaron al menos una medida, frente al 37,7% de las de ISP. La modificación más frecuente en ambos fue la de la ficha técnica y del inserto del producto. Sin embargo, el ISP incluyó el monitoreo continuo de metilfenidato y diacereína (por riesgo de priapismo y diarrea severa), así como de la vacuna ChAdOx1 contra COVID-19 (por posible síndrome de Guillain-Barré y fuga capilar sistémica). Además, requirió un plan de gestión de riesgos para medios de contraste con gadolinio debido a su acumulación cerebral ^13^. La DIGEMID no reportó requerimientos de monitoreo continuo, medidas adicionales de farmacovigilancia ni retiros preventivos de medicamentos.

Las categorías de recomendaciones clínicas más frecuentes fueron «Monitorear, evaluar, informar al paciente; considerar la dosis y poblaciones vulnerables/específicas» y «Monitorear, evaluar y, si es necesario, suspender el tratamiento». También se registraron restricciones de uso: en Perú, la lidocaína tópica para la dentición pediátrica fue limitada en 2015 debido a riesgos graves (convulsiones y muerte), y en Chile, el almidón hidroxietílico fue restringido en 2018 por su asociación con daño renal y una mayor mortalidad en pacientes con septicemia.

## DISCUSIÓN

Entre 2014 y 2023, la DIGEMID emitió 100 NSPF y el ISP 53, aproximadamente 0,83 por mes en Perú y 0,44 por mes en Chile. Se observaron picos en 2014 y 2018, seguidos de una disminución desde 2019, que en Perú alcanzó cero en 2022. Esta reducción podría relacionarse con la pandemia de COVID-19, que reorientó los recursos regulatorios hacia el control de calidad y la vigilancia de los medicamentos falsificados ^14^^,^^15^.

En Perú, las NSPF se centraron principalmente en RAMs dermatológicas (SSJ, NET), mientras que en Chile predominaron la hepatotoxicidad y el cáncer. Estas diferencias podrían explicarse por variaciones en los patrones de uso de medicamentos, en la farmacovigilancia y en factores poblacionales. En Perú destacaron los inhibidores SGLT-2 y en Chile los inhibidores DPP-4, reflejando su creciente uso en diabetes tipo 2. Este incremento se ha asociado con un aumento de RAMs reportadas, como infecciones genitales, cetoacidosis y eventos raros con SGLT-2, y pancreatitis e hipersensibilidad con DPP-4 ^16^^,^^17^, lo que resalta la necesidad de fortalecer la farmacovigilancia activa.

Se observaron diferencias en las NSPF entre Perú y Chile según la clasificación ATC, lo que podría reflejar variaciones en prácticas terapéuticas y prioridades de seguridad. En Perú, la mayoría de las NSPF se centró en medicamentos del sistema nervioso (N) (22%), incluyendo antidepresivos, antipsicóticos y anticonvulsivos, asociados a RAMs dermatológicas, neurológicas y psiquiátricas, lo que coincide con lo reportado en otros países ^7^. En Chile, predominó el grupo Tracto Alimentario y Metabolismo (A) (19%), que incluye fármacos para trastornos gastrointestinales y metabólicos como la diabetes tipo 2 y la obesidad. Estas diferencias podrían reflejar variaciones en la carga de enfermedad y en los patrones de tratamiento, subrayando la necesidad de una farmacovigilancia adaptada al contexto local.

Al evaluar la presencia de los medicamentos descritos en las NSPF en el LNME, el 46% de los PF en Perú y el 27% en Chile estaban incluidos en sus respectivos listados. Si bien los medicamentos esenciales son seguros y efectivos su uso extendido incrementa la probabilidad de RAMs, lo que hace indispensable su monitoreo continuo ^9^^,^^18^. El LNME peruano incluye actualmente 434 PF, de los cuales cuatro están bajo farmacovigilancia intensiva ^9^. Nuestros hallazgos identificaron otros medicamentos esenciales con potencial de causar RAMs graves, por lo que recomendamos que la DIGEMID amplíe sus programas de farmacovigilancia activa para incluir fármacos de alto riesgo y alto consumo.

Las NSPF emitidas por DIGEMID e ISP se basaron en alertas de la FDA y, solo en Perú, se identificó una alerta sustentada en la farmacovigilancia local. Esto podría sugerir limitaciones en los sistemas locales de farmacovigilancia, principalmente, para generar señales de seguridad a partir de datos nacionales. Asimismo, la evidencia que sustenta las alertas internacionales proviene de notificaciones espontáneas de RAMs y estudios observacionales poscomercialización. Aunque los ensayos clínicos son clave para evaluar eficacia, los estudios farmacoepidemiológicos y los reportes de RAMs son fundamentales para detectar riesgos en poblaciones amplias ^19^^,^^20^. Esto resalta la necesidad de fortalecer los sistemas de reporte e incorporar estudios farmacoepidemiológicos en Perú y Chile, con el fin de mejorar la toma de decisiones regulatorias y la seguridad del paciente.

En cuanto a las acciones regulatorias, aunque en ambos países predominó la modificación de la ficha técnica e inserto del producto, el ISP implementó medidas adicionales, como el monitoreo continuo de fármacos específicos (metilfenidato y vacunas como ChAdOx1 y Ad26.COV2) y la solicitud de planes de gestión de riesgos para el gadolinio debido a su posible acumulación cerebral. Estas acciones reflejan un enfoque más preventivo y proactivo que el de DIGEMID, que tiende a actuar de forma más reactiva, respondiendo a eventos ya ocurridos o a alertas externas. Estas diferencias podrían explicarse por variaciones en los marcos regulatorios, en el acceso a la evidencia y en la disponibilidad de recursos.

Este estudio presenta algunas limitaciones. Las NSPF, al ser documentos concisos, no incluyen todas las disposiciones técnicas (ej. informes periódicos de seguridad o planes de gestión de riesgos), lo que podría subestimar las acciones regulatorias. Además, no fue posible determinar si los retiros de PF fueron definitivos o temporales. No obstante, el estudio tiene fortalezas relevantes: es, hasta donde sabemos, el primer análisis descriptivo-comparativo de NSPF en países latinoamericanos, que evalúa no solo la frecuencia de alertas, sino también la trazabilidad de la evidencia (internacional vs. local) y la inclusión en los LNME, ofreciendo una visión integral de la dependencia de la vigilancia internacional y de su impacto en la salud pública regional.

Entre 2014 y 2023 se emitieron 153 NSPF relacionadas con RAMs, con un promedio de aproximadamente dos por mes en Perú y una en Chile. Se identificaron diferencias en su contenido, particularmente en las RAMs y la distribución por ATC. La mayoría de las NSPF se basaron en alertas de la FDA, y la modificación de la ficha técnica fue la principal medida regulatoria. No obstante, el ISP implementó acciones adicionales, como el monitoreo continuo, las restricciones de uso y los planes de gestión de riesgos. Estos hallazgos evidencian la necesidad de fortalecer la farmacovigilancia local en ambos países, especialmente la notificación espontánea de RAMs.
